# Diagnosis and Molecular Profiles of Large Cell Neuroendocrine Carcinoma With Potential Targets for Therapy

**DOI:** 10.3389/fonc.2021.655752

**Published:** 2021-07-07

**Authors:** Helmut Popper, Luka Brcic

**Affiliations:** Diagnostic and Research Institute of Pathology, Medical University of Graz, Graz, Austria

**Keywords:** pulmonary neuroendocrine tumors, SCLC, LCNEC, mutations, subtyping

## Abstract

Large cell neuroendocrine carcinoma (LCNEC) together with small cell carcinoma (SCLC) and typical and atypical carcinoids form the group of pulmonary neuroendocrine tumors. LCNEC and SCLC are high-grade carcinomas. Although both can be found outside the thoracic cavity, they are most common in the lung. LCNEC differs from SCLC by morphologic pattern, and by cytological features such as nuclear size, nucleoli, chromatin pattern, but also by genetic differences. Originally thought to represent a single entity, it became evident, that three subgroups of LCNEC can be identified at the molecular level: a SCLC-like type with loss of retinoblastoma 1 gene (RB1) and TP53 mutations; a non-small cell lung carcinoma (NSCLC)-like type with wildtype RB1, TP53 mutation, and activating mutations of the phosphoinositol-3 kinase (PI3K-CA), or loss of PTEN; and a carcinoid-like type with MEN1 gene mutation. These subtypes can be identified by immunohistochemical staining for RB1, p53, and molecular analysis for PI3K and MEN1 mutations. These subtypes might also respond differently to chemotherapy. Immuno-oncologic treatment has also been applied to LCNEC, however, in addition to the evaluation of tumor cells the stroma evaluation seems to be important. Based on personal experiences with these tumors and available references this review will try to encompass our present knowledge in this rare entity and provoke new studies for better treatment of this carcinoma.

## Introduction

Large cell neuroendocrine carcinoma (LCNEC) was originally created during a study of atypical carcinoids (ATC) with an unusual dismal outcome (1). The major criteria were a neuroendocrine morphology with rosettes and trabecules, the expression of neuroendocrine markers, such as chromogranin A (CGA), synaptophysin (SYN), neural cell adhesion molecule (CD56/NCAM), and others. In contrast to carcinoids, LCNEC presented with large nuclei (> 26µm), coarse chromatin, and frequently enlarged nucleoli. The mitotic rate was above 10/2mm^2^, and large necrotic areas are frequently seen. The prognosis for this group of carcinomas was similar to that of small cell lung carcinoma (SCLC), which is also a high-grade neuroendocrine carcinoma (2–4). The differentiation between SCLC and LCNEC is usually based on morphology: nuclei 17-23µm, absence of nucleoli, dense heterochromatin in SCLC; nuclear size >26µm, coarse chromatin, and frequently enlarged nucleoli in LCNEC. Later on, other carcinomas with large cell morphology and expression of neuroendocrine markers in > 10% of tumor cells were added to the LCNEC category (in previous WHO classifications this was based on 25% of tumor cells) – in these cases, a classical neuroendocrine morphology was not always present, but large areas of necrosis were seen. Whereas the low and intermediate-grade carcinoids arise from neuroendocrine precursor cells, and precursor lesions such as tumorlets can often be seen, the high grades carcinomas SCLC and LCNEC arise from undifferentiated probably stem cell-like precursors, but in LCNEC a transition from atypical carcinoid to LCNEC is suspected (5–8). The great majority of high-grade carcinomas are associated with cigarette smoking (9, 10). In a previous study we have shown similarities and differences in chromosomal alterations between SCLC and LCNEC. Losses of 3p, 4q, 5q, and 13q and gains of 5p were common in both entities. A gain of 3q and losses on chromosome 10 were frequently seen in SCLC but not in LCNEC. Gains of 6p occurred more frequently in LCNEC (11).

From the beginning treatment for LCNEC was discussed and handled controversially for several decades, preferring either a non-small cell lung carcinoma (NSCLC) protocol including cisplatinum or a SCLC-based protocol (12, 13).

## Methods and Material

In our lung pathology archive 13412 carcinomas were collected between 1986 and 2012. Of these 163 were diagnosed as LCNEC. In addition to a neuroendocrine morphology also immunohistochemistry for the markers γγ-enolase (NSE), gastrin-releasing peptide (GRP), CGA, NCAM, SYN, calcitonin (CT), vasoactive intestinal peptide (VIP), neural peptide Y (NPY), adrenocorticotropin (ACTH), and PGP9.5, respectively, combined with low-molecular weight cytokeratin antibodies had to be applied in 53 cases to reach a definite diagnosis*. In 25 cases a mixed SCLC/LCNEC diagnosis was made. Other combinations with LCNEC restricted to few cases were squamous cell carcinoma, adenocarcinoma, and large cell carcinoma.

(*During decades markers for neuroendocrine differentiation have changed; NSE was one of the earliest markers used in this respect, whereas now CGA, NCAM, and SYN are most often used)

## Morphology and Diagnosis

Clinically LCNEC presents as a tumor mass on CT scan and X-ray. There are no specific clinical symptoms. On gross examination the only feature that might point to LCNEC are large areas of necrosis, which by themselves are not specific.

**Figure 1:**
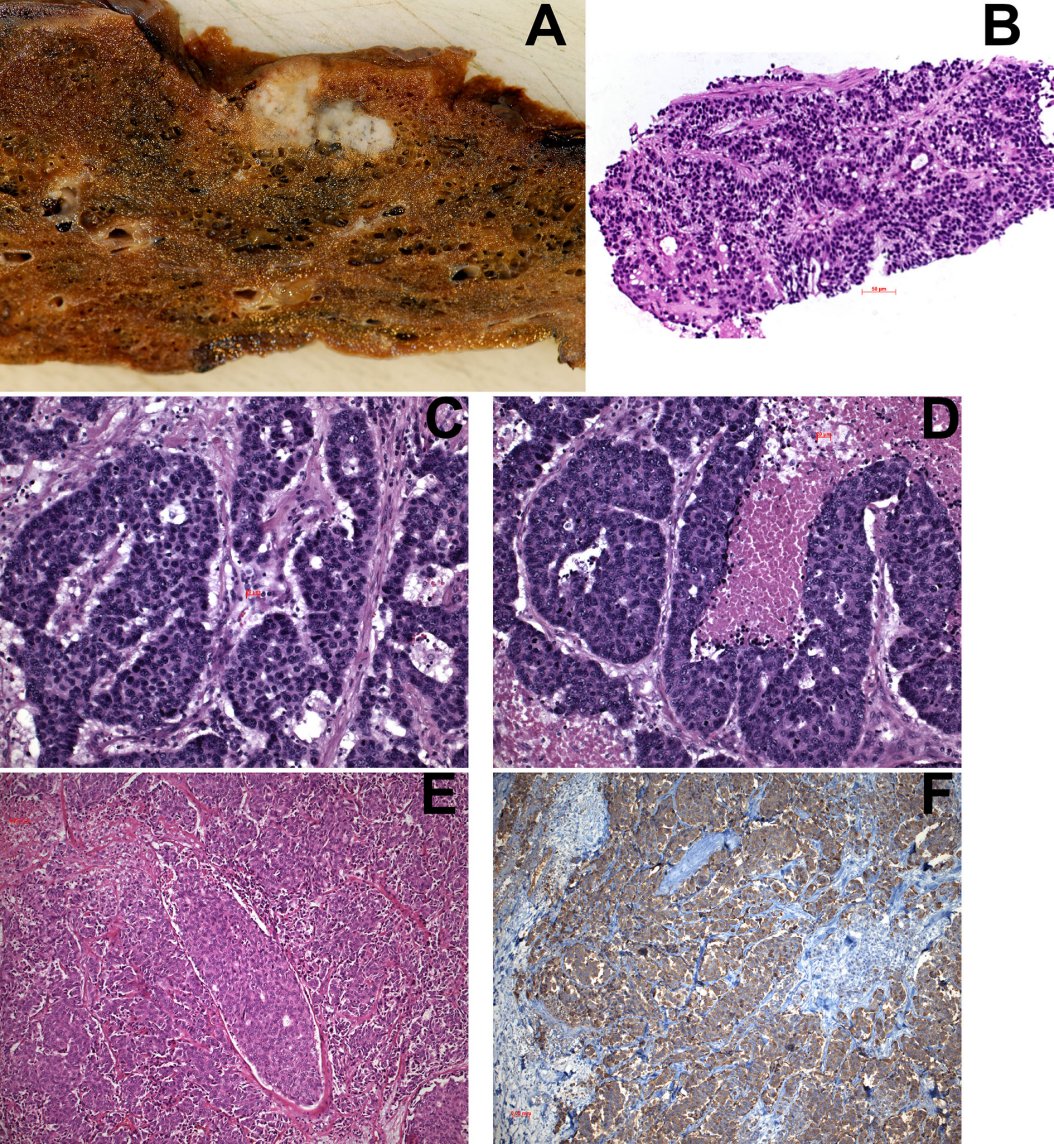
**(A)** Macroscopy of LCNEC, showing two nodular lesions separated by a small bridge of parenchyma; areas of necrosis are seen on the left side. **(B)** transthoracic needle biopsy of a LCNEC. **(C, D)** Examples of LCNEC with neuroendocrine morphology (rosettes) and areas of necrosis in **(D)**. **(E)** Another LCNEC without neuroendocrine morphology. **(F)** Immunohistochemistry in this case showed positivity for CGA in almost 100% of tumor cells. H&E and IHC for CGA, magnifications x70, x200, and x400.

Morphologically LCNEC is defined by a neuroendocrine pattern, i.e., rosettes, trabecules, and solid cell nests (**Figure 1**). However, the neuroendocrine morphology is not always clearly visible, and in some cases only nesting of tumor cells is present. On low-power view, LCNEC looks organoid, similar to a carcinoid, but on higher magnification abundant mitoses are obvious. Nuclei are large polymorphic, 25-35 µm, with coarse, granular chromatin, enlarged and prominent nucleoli, landscape-like necrosis is usually present. To confirm the diagnosis staining for neuroendocrine markers (NCAM, SYN, CGA, PGP9.5) is recommended: at least 10% of cells should be positive with at least one neuroendocrine marker. LCNEC in comparison with SCLC produces and less secretes often hormones. It is also positive for low-molecular weight cytokeratin. In our cases, the vast majority presented with mitotic counts > 35/2mm^2^, however, there were few cases with a neuroendocrine morphology with mitotic counts from 11-18/2mm^2^. Based on the WHO classification (14) these cases had to be classified as LCNEC. However, we hypothesize, that likely these cases behave more like atypical carcinoids or in between ATC and LCNEC. Only one patient of our cases died within 3 years, all the others had an overall survival beyond 5 years. These cases all had metastasis in N2 lymph nodes but with low T stage (T1 or T2) and no metastasis outside the lung. Quinn et al. published a series of cases with similar findings, although mitotic counts in their cases ranged from 11-61/2mm^2^ (15). As these cases are rare, a multi-institutional investigation is needed to evaluate these cases and to position them into the classification of neuroendocrine tumors.

## Heterogeneity of LCNEC

To better define LCNEC, Rekhtman and colleagues analyzed 45 cases of LCNEC by next-generation sequencing. They identified two large and one small groups of cases, characterized by a specific genomic profile. One was a SCLC-like group with mutations of TP53 and loss of RB1, and other alterations such as MYC-L amplification. Another NSCLC-like group presented with TP53, KRAS, STK11/LKB1, and KEAP1 mutations and retained RB1, and also frequent alterations of NOTCH family genes. The third group presented with a carcinoid-like morphology and MEN1 gene mutation (16). Unfortunately, these results were not correlated with mitotic counts. These findings were also confirmed in the study by Miyoshi et al. Here 78 LCNEC samples were sequenced for the coding exons of 244 cancer-related genes. Inactivating mutations were seen for TP53 and RB1, but the mutation frequency in RB1 was lower than in SCLC. Other genetic alterations were detected in the PI3K/AKT/mTOR pathway and activating alterations were detected in KRAS, FGFR1, KIT, ERBB2, HRAS, and EGFR (17). Deleting the tumor suppressors, RB1 and PTEN, and deactivating TP53, Lazaro and colleagues created a mouse model for LCNEC, confirming again the importance of RB1 and PTEN in the development of this carcinoma (18). In two different studies, Simbolo et al. identified three different LCNEC clusters: cluster 1 with inactivation of TP53 and RB1 with an absence of MEN1 mutations; cluster 2 with mutations of TP53, MEN1, and RB1 mutations; and cluster 3 without RB1 alterations but frequent MEN1 and TP53 mutations. These findings were also evaluated and confirmed by immunohistochemistry. Patients in cluster 1 had shorter cancer-specific survival than all others (19). By performing whole-exome sequencing for 418 genes in carcinoids, LCNEC, and SCLC, the authors found MEN1 alterations almost exclusively in carcinoids, whereas TP53 and RB1 alterations were present in the high-grade carcinomas. Chromatin-remodeling genes, such as histone modifiers and members of SWI-SNF complexes, were seen at similar rates in all neuroendocrine tumors (20). In another carcinoid-study, carcinoids showed MEN1 gene alterations, resulting in failures of chromatin remodeling, while LCNEC were characterized by mutations in DNA repair genes (loss of orthopedia homeobox) and upregulation of the RET gene. These authors also reported on one group with biallelic inactivation of TP53 and RB1, and the second with biallelic inactivation of TP53 and mutations of the serine/threonine kinase 11 gene (STK11) and kelch like ECH associated protein 1 gene (KEAP1) (21).

The neuroendocrine phenotype has been attributed to the neuroendocrine master regulator ASCL1/hASH (22, 23). However, the context of cells where ASCL1 is expressed is important: in the wrong context, the expression will create different types of carcinomas expressing neuroendocrine markers, but not LCNEC or SCLC (24, 25). This mechanism might probably explain the trans-differentiation of EGFR-mutated adenocarcinomas into SCLC- or LCNEC-like carcinoma types. Here the antagonism of NOTCH/Hes1 and ASCL1 come into play: Inactivation of the NOTCH-Hes1 axis might result in overexpression of ASCL1 and pave the way to a neuroendocrine phenotype (26). In the meanwhile, other regulators of neuroendocrine differentiation have been identified in SCLC as well as LCNEC. NeuroD was identified as another neuroendocrine master gene, but less frequently in LCNEC. Interestingly patients with NeuroD expression had better survivals (27). Similar to NeuroD, ASCL1 was found to be less often expressed in LCNEC, which might explain the difficulties in staining patterns in this category of high-grade neuroendocrine carcinoma (28). Another interesting observation and probably therapy relevant was a mutation of the NTRK2 and NTRK3 genes reported by Marchetti (29). In contrast to a rearrangement seen in several malignancies, here an activating mutation prevails. If this can be treated by NTRK inhibitors needs to be proven.

## Diagnosis in Small Biopsies

In small biopsies LCNEC can be diagnosed, if rosettes and trabecules are present (**Figure 1B**), and the nuclei are large (diameter > 26µm), the chromatin is coarse, with prominent, middle-sized nucleoli. High mitotic counts might be encountered, whereas the large necrotic areas might not be seen. Immunohistochemistry should be done using a panel of at least two neuroendocrine markers. In cytological preparations the diagnosis is more difficult, because cell adhesion is much less compared to carcinoids, which results in rarely seen rosettes. If the nuclear features are present and numerous mitoses are seen, an immunocytochemistry for neuroendocrine markers should be performed.

## Aspects for a Therapy

**Figure 2:**
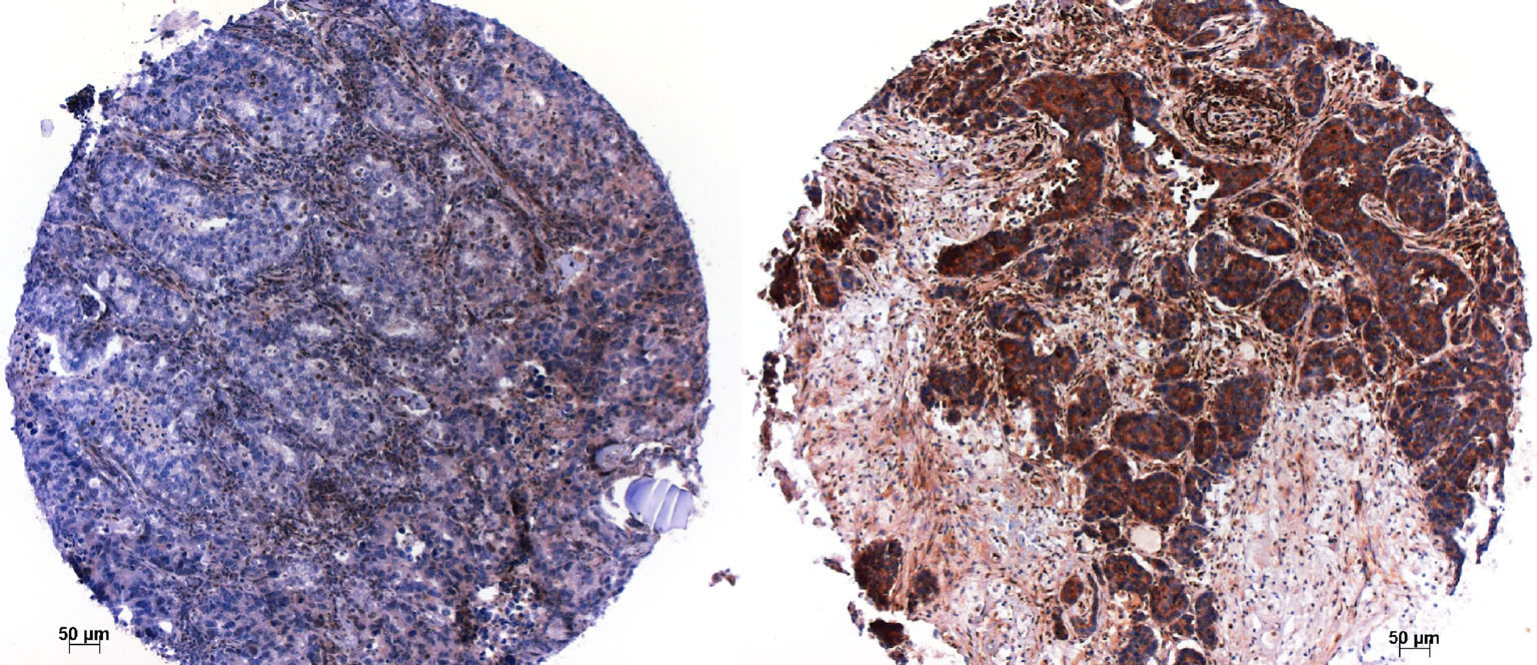
Immunohistochemistry for RB1; a case, which lost RB1 on the left, and another case, which retained RB1 to the right. Magnification x100.

The prognosis of patients with LCNEC is similar to SCLC. Surgery is recommended for LCNEC in clinical stages I to IIIA. The discussion of which type of chemotherapy has to be applied remained controversial for decades (12, 13, 30–32). In recent times a chemotherapy regimen similar to SCLC is favored (31, 33). In a meta-analysis by He J. et al. overall and progression free survival was found to be superior, if LCNEC patients received a chemotherapy regimen similar to SCLC protocols (34) However, recurrence and metastasis are as high as in SCLC (35). The subtyping of LCNEC has opened possibilities for stratification in therapy: LCNEC, which have lost RB1 have been shown to respond to SCLC-like chemotherapy, whereas those retaining RB1 (**Figure 2**) and having either loss of PTEN, activating mutation of PI3KCA, combined with mutations of TP53, respectively, respond better to cisplatin chemotherapy (33, 36). Other therapeutic targets are being identified in LCNEC: A NTRK2/3 mutation has been reported, which might be targeted by NTRK-inhibitors (29). Furthermore, a FGFR2 mutation was detected exclusively in LCNEC (37), for which tyrosine kinase inhibitors are available. Four clinical studies targeting FGFR1 and FGFR2 are either closed or ongoing, however including so far only pulmonary squamous cell carcinoma (NCT 03762122, NCT01795768, NCT02965378, NCT01004224). Recently a new treatment was tested for SCLC. A toxin coupled to DLL3 expressed on SCLC cells showed some promises in phase 2 studies, however, failed in phase 3. DLL3 is also expressed in the majority of LCNEC, irrespective of RB1 (38). If a similar approach from SCLC might be used also in LCNEC deserves further studies (39). Resistance to chemotherapy and radiotherapy is a frequent event in both high-grade neuroendocrine carcinomas. Tumor-associated macrophages (TAMs) play an important role in this respect. The receptor tyrosine kinases Tyro3, Axl, and MerTK on macrophages are important in regulating these TAMs. These receptors help in polarizing macrophages into tumor-friendly M2 types (40). Using cell lines Ramkumar et al. inhibited Axl with a small molecule BGB324 and induced inhibition of cell proliferation and DNA damage in NSCLC and LCNEC (41). This might increase our repertoire for tumors developing resistance to radiotherapy and chemotherapy.

Another option for the treatment of LCNEC is immunotherapy (**Table 1**). PD-L1 expression in LCNEC was associated with poor survival, while PD-L1 expression in the tumor microenvironment seemed to have a beneficial effect (42). Other studies on the expression of PD-L1 in both SCLC and LCNEC were performed, and several studies analyzed not only the expression of PDL1 on tumor cells but also on cells of the tumor stroma. The frequency of PD-L1 expression on tumor cells was in the range of 15-35% (43–47). However, more important than the expression on tumor cells was the expression on stromal cells and the association with infiltrating cytotoxic lymphocytes (CD8+) (43). Furthermore, analyzing lymphocytes for additional markers, Ohtaki et al. showed a favorable prognosis for cases with FoxP3 expressing tumor-associated lymphocytes, whereas the presence of CD4+ helper lymphocytes conferred an unfavorable prognosis (48). This was confirmed by the study of Shirasawa et al, who also included lymphocyte density into their study (49). Combining immunotherapy with chemo- and radiotherapy was shown to improve survival (50). Trials on immunotherapy are still ongoing (**Table 2**). Recently the stimulator of interferon genes (STING) has been shown as a probably new target to stimulate the patients’ immune system towards cancer. Normally STING is activated, if DNA or RNA is detected within the cytoplasm of cells. Treatment with cisplatin increases fragmented DNA and stimulates the STING pathway in STK11 and TP53 co-mutated NSCLC and LCNEC (51). In these cases, a PD-L1 therapy could be successfully applied.

**Table 1:** Studies reporting on treatment modalities for LCNEC; TILs, tumor associated lymphocytes; TMB, tumor mutational burden.

Authors	treatment
Rossi et al	PDGFRa/b, MET, chemotherapy
Igawa et al	chemotherapy
Eichhorn et al	PL-L1 immunotherapy
Arpin et al	PD-L1 immunotherapy
Dudnik et al	PD-L1 immunotherapy
Hermans et al	PD-L1 immunotherapy
Tsuruoka et al	PD-L1 immunotherapy
Kim et al	PD-L1 immunotherapy, TILs, TMB
Ohtaki et al	PD-L1 immunotherapy, TILs
Shirasawa et al	PD-L1 immunotherapy
Komiya et al	PD-L1 immunotherapy combined chemo- and radiotherapy
Della Corte et al	PD-L1 immunotherapy and STING pathway activation

**Table 2:** Ongoing clinical trials focusing on immunotherapy (chemotherapy trials are not included here).

Clinical trials	
NCT03976518	Atezolizumab in NSCLC with rare histologies, phase II
NCT03901378	Pembrolizumab in GI-tract and Lung LCNEC, withdrawn
NCT02834013	Nivolumab and Ipilimumab in rare tumors; including LCNEC but also salivary gland tumors; recruiting
NCT03591731.	Nivolumab and Ipilimumab in GI-tract and Lung LCNEC, recruiting
NCT03305133	Evaluation of PD-L1 expression in LCNEC, completed
NCT03728361	Nivolumab and temozolomide in refractory SCLC and advanced neuroendocrine cancer, phase II recruiting

Some of the above-mentioned biomarkers, like RB1 and p53 expression, can easily be evaluated by immunohistochemistry (**Figure 2**). As next-generation sequencing is regularly done in many laboratories for NSCLC with non-squamous histology, it should not be a problem to establish a mutational profile (including the above-mentioned genes) for LCNEC cases as well.

In conclusion, immunohistochemistry and molecular profiling will complement histology for better diagnostic definition and prognostic stratification of lung neuroendocrine tumors, and especially LCNEC, and will open new avenues for treatment. The molecular characterization of LCNEC should be included in the routine pathology practice.

## Author Contributions

HP designed the review. LB and HP worked on the manuscript and finalized it. All authors contributed to the article and approved the submitted version.

## Conflict of Interest

The authors declare that the research was conducted in the absence of any commercial or financial relationships that could be construed as a potential conflict of interest.
